# Did southern Western Ghats of peninsular India serve as refugia for its endemic biota during the Cretaceous volcanism?

**DOI:** 10.1002/ece3.603

**Published:** 2013-08-12

**Authors:** Jahnavi Joshi, Praveen Karanth

**Affiliations:** Centre for Ecological Sciences, Indian Institute of ScienceBangalore, 560012, India

**Keywords:** Centipedes, historical biogeography, invertebrates, species richness, time tree, tropics

## Abstract

The Western Ghats (WG) of south India, a global biodiversity hotspot, has experienced complex geological history being part of Gondwana landmass and encountered extensive volcanic activity at the end of Cretaceous epoch. It also has a climatically and topographically heterogeneous landscape. Thus, the WG offer a unique setting to explore the influence of ecological and geological processes on the current diversity and distribution of its biota. To this end, three explicit biogeographical scenarios were hypothesized to evaluate the distribution and diversification of wet evergreen species of the WG – (1) southern WG was a refuge for the wet evergreen species during the Cretaceous volcanism, (2) phylogenetic breaks in the species phylogeny would correspond to geographic breaks (i.e., the Palghat gap) in the WG, and (3) species from each of the biogeographic subdivisions within the WG would form distinct clades. These hypotheses were tested on the centipede genus *Digitipes* from the WG which is known to be an ancient, endemic, and monophyletic group. The *Digitipes* molecular phylogeny was subjected to divergence date estimation using Bayesian approach, and ancestral areas were reconstructed using parsimony approach for each node in the phylogeny. Ancestral-area reconstruction suggested 13 independent dispersal events to explain the current distribution of the *Digitipes* species in the WG. Among these 13 dispersals, two dispersal events were at higher level in the *Digitipes* phylogeny and were from the southern WG to the central and northern WG independently in the Early Paleocene, after the Cretaceous Volcanism. The remaining 11 dispersal events explained the species’ range expansions of which nine dispersals were from the southern WG to other biogeographic subdivisions in the Eocene-Miocene in the post-volcanic periods where species-level diversifications occurred. Taken together, these results suggest that southern WG might have served as a refuge for *Digitipes* species during Cretaceous volcanism.

## Introduction

A strong comprehensive framework to identify the underlying evolutionary processes operating on contemporary species dynamics is emerging (Harrison and Grace 2007; Ricklefs 2007). Many recent theoretical and empirical studies have highlighted the role of historical biogeography in explaining current geographic patterns of biodiversity (Harrison and Grace 2007; Ricklefs 2007; Wiens 2007; Leibold et al. 2010). Tropical forests with their stunning diversity have especially attracted the attention of many researchers (Schneider et al. 1999; Moritz et al. 2000; Haffer 2008; Vences et al. 2009; Hoorn et al. 2010). In this regard, much needs to be done with respect to understanding species dynamics and biogeography of the Western Ghats (WG) of tropical South Asia, which has been identified as a global biodiversity hotspot because it harbors high levels of biodiversity and endemicity (Myers et al. 2000). WG of peninsular India (PI) have also experienced complex geological history (Briggs 2003). Here, we present a brief review of the geological and climatic history of the WG and then propose three biogeographic scenarios for the wet evergreen species of the WG with explicit predictions.

### Western Ghats

**Figure 1:**
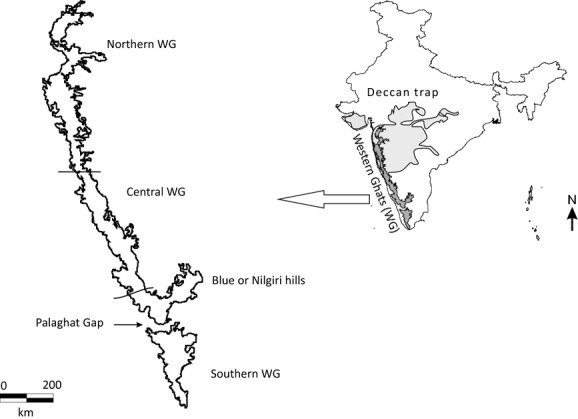
A map of India showing Western Ghats with approximate boundaries of Deccan trap and the four biogeographic zones.

The WG is a chain of mountains running along the west coast of PI for over 1600 km (8° to 21°N) with one major low-elevation break called the Palghat Gap (PG) (Fig. 1) (Subramanyam and Nayar 1974; Ali and Ripley 1987). In addition to high endemicity, WG exhibits substantial heterogeneity in vegetation and landscape types, as well as a distinct north–south gradient in seasonality and rainfall (Pascal 1988). Based on plant species composition, WG has been divided into four zones, namely (1) northern WG (NWG), (2) central WG (CWG), (3) Nilgiri or Blue Mountains, and (4) southern WG (SWG) (Fig. 1) (Subramanyam and Nayar 1974).

Interestingly, species richness and endemicity are not distributed uniformly across the WG. Studies on plants from across the WG have revealed that the southern parts of the WG are extremely diverse and have high endemicity compared with the central and NWG (Pascal 1988; Gimaret-Carpentier et al. 2003; Pascal et al. 2004; Davidar et al. 2007). Additionally, birds, amphibians, and fishes also exhibit similar diversity and endemicity patterns, that is, the SWG being more diverse with a higher proportion of endemics than the central and NWG (Dahanukar et al. 2004; Aravind and Gururaja 2010). Most of these studies have identified contemporary ecological factors like seasonality, productivity, climate, and short dry period length as the processes governing the aforementioned patterns. However, speciation and biogeographic processes are poorly understood for the WG biota.

As the WG is embedded in PI, its geological and climatic history is tied to that of PI. PI was dominated by warm tropical climate during the Late Cretaceous period (Singh et al. 2006; Samant and Mohabey 2009). Toward the end of the Cretaceous (around 70–65 mya), PI experienced extensive volcanic activity that resulted in the formation of the Deccan traps (Singh et al. 2006; Samant and Mohabey 2009). The Deccan traps are one of the largest continental flood basalts in the world and are confined to the northern parts of PI. This volcanic activity that spanned between 0.5 and 5 million years (my) has been speculated to have triggered mass extinctions in PI and elsewhere (Samant and Mohabey 2009). Recent pollen analyses from western and north-western parts of PI indicate the reestablishment of wet evergreen forests in the Early Paleocene after the extensive volcanic activity (Prasad et al. 2009). Thus, SWG may have served as refugia for wet evergreen plant species that were once widely distributed during the prevolcanic period. Another study on diversity and distribution of wet evergreen plants also discussed the plausibility of southern parts of the WG being a refuge for the wet evergreen plant species (Gimaret-Carpentier et al. 2003). Today, wet evergreen forests are one of the most dominant forests types in the WG and are confined predominantly to the western escarpment of this mountain range (Pascal 1988).

### Biogeographical scenarios for WG

Given that SWG might have served as refugia for wet evergreen forest species during periods of volcanism, the advent of suitable conditions in the post-volcanic period could lead these species to have dispersed from SWG to central and NWG. This scenario (S1) can be tested in a historical biogeographical framework that uses molecular phylogenies in conjunction with molecular dating and event-based biogeographical analysis. If this scenario was true, then in the phylogeny of a group of WG endemics, the CWG and NWG species would be nested within a clade constituting SWG species. Additionally, the ancestral areas of the deeper nodes in the phylogeny should be in SWG and dispersals from SWG into CWG and NWG should fall in the post-volcanic period. Recent molecular phylogenetic studies on the amphibians and centipedes of the WG reported ancient lineages in the SWG (Biju and Bossuyt 2003; Bossuyt et al. 2004; Roelants et al. 2007; Joshi and Karanth 2011a), but these studies did not speculate or test the SWG refugia hypothesis, which is being presented here for the first time.

Alternately, physiographic or ecological barriers might have played an important role in the current distribution of species. As mentioned earlier, the WG is interrupted at 11°N by a 30-km wide gap called the PG. Distribution data of plants, birds, dragonflies, arboreal frogs (*Philautus*), and fishes suggest that this gap might have served as a potential geographical barrier (Fraser 1936; Subramanyam and Nayar 1974; Ali and Ripley 1987; Dahanukar et al. 2004; Biju et al. 2005). Few molecule-based biogeographic studies have assessed the role of the PG in shaping current distribution of flora and fauna. Microsatellite data have shown strong population structuring across the gap in the case of Asian Elephants (Vidya et al. 2005). For plants, like *Eurya nitida* and *Gaultheria fragrantissima*, PG emerged as an important barrier structuring populations within the species across the gap (Bahulikar et al. 2004; Apte et al. 2006). It also appears to be an important barrier for the high-elevation endemic bird, the White-bellied Shortwing (*Brachypteryx major*) (Robin et al. 2010). Thus, these studies suggest that PG might have served as a barrier to gene flow in species distributed on either side of the gap. However, all these studies have focused on intraspecific variation, and the role of the PG in shaping interspecific patterns of distribution needs to be evaluated. Interestingly, a study on a species of a group of caecilians, traditionally considered to have limited dispersal ability, showed that the gap did not function as a barrier for the species (Gower et al. 2007). Nevertheless, results from a molecular work which included multiple taxonomic groups from India and Sri Lanka pointed toward the role of PG in shaping species assemblages (Bossuyt et al. 2004). Thus, if PG is indeed a major biogeographical barrier, then the distributions of either sister species or clades in a phylogeny would not overlap and would fall on either side of the gap (Scenario 2, S2).

In addition to the PG, the four biogeographic subdivisions of the WG might have also restricted species movements and they serve as ecological barriers. These subdivisions are spatially separated and differ in their climatic envelope and floral composition (Subramanyam and Nayar 1974) (also see Fig. 1). Thus, species in each subdivision might be more closely related to each other than to species from other subdivisions. This scenario (S3) would be reflected in the phylogenetic tree by species from each subdivision forming distinct clades. To date, there is no published molecular work that addresses this ecological barrier scenario for WG.

Three explicit biogeographical scenarios are presented to explain current distribution of flora and fauna in the WG. These hypotheses can be used to generate specific predictions which can then be tested in a phylogenetic framework using biogeographic methods. These scenarios need not be mutually exclusive as they might have operated simultaneously at same/different spatial and temporal scales. To test the predictions of the above-mentioned hypotheses, the centipede genus *Digitipes* (Scolopendridae: Otostigmini) of the WG was selected. A recent molecular phylogenetic study on scolopendrid centipedes established the monophyly of Indian *Digitipes* species (Joshi and Karanth 2011a) and another study on species delimitation proposed a comprehensive phylogenetic hypothesis along with revised distribution maps (Joshi and Karanth 2011b). Current distribution patterns of *Digitipes* species in the WG suggest that SWG is more diverse and has more endemic species than CWG and NWG. Specifically, the SWG have five species of which three are endemics, Nilgiri hill range has three species with no endemics and the CWG has three with one endemic species, whereas the NWG has one nonendemic species (Joshi and Karanth 2011b). Furthermore, most of the species are found at lower (>500 mean sea level [msl]) and midelevation forests (500–1500 msl), except for one in the high-elevation areas of the WG (<1500 msl) (Joshi and Karanth 2011b). Additionally, diversification in the *Digitipes* lineage started during the Late Cretaceous when the peninsular Indian plate was on its northward journey, and experienced long periods of isolation (Joshi and Karanth 2011a). Thus, *Digitipes* of WG is ideally suited to test the biogeographical scenarios presented above as they represent an ancient endemic lineage (originating in the pre-volcanism period) and also exhibits a trend of decreasing species richness and endemicity with increasing latitude.

To this end, a recently published *Digitipes* phylogeny was used to estimate the divergence dates via a Bayesian approach. Additionally, historical biogeographic analysis was performed on the *Digitipes* phylogeny to reconstruct ancestral areas and test biogeographic hypotheses.

## Materials and Methods

### Divergence time estimation

Sequences of two mitochondrial DNA (mt) markers, the 16S ribosomal gene (16S) and cytochrome c oxidase I (COI), belonging to seven species from a recently published phylogeny of the genus *Digitipes* were used for the divergence dating analyses(Joshi and Karanth 2011b). The program BEAST (v1.4.8), which uses a Bayesian approach and Markov chain Monte Carlo (MCMC) method (Drummond and Rambaut 2007), was implemented on the partitioned mtDNA dataset to estimate node ages. A previous study on the diversification of scolopendrids showed that the genus *Digitipes* started diversifying ∼86 ± 20 mya in the WG (Joshi and Karanth 2011a). This date was used as a calibration point in this study to date species-level diversification in the phylogeny. A normally distributed prior with mean of 86 my was applied to the most recent common ancestor (tMRCA) for the in-group comprising all *Digitipes* species. The members of the genus *Rhysida* were used as out-group (Joshi and Karanth 2011a). The model and priors settings for the analyses were as follows: the GTR + I model of sequence evolution with uniform priors was applied on the partitioned dataset as suggested by Model test (Posada and Crandall 1998). A relaxed molecular clock model with an uncorrelated lognormal distribution was used (Drummond et al. 2006). Additionally, the Yule speciation process, which is considered to be appropriate for species-level phylogenies, was used. The program was run for 100 million generations and convergence of the chains to the stationary distribution was determined using the program TRACER (v1.4.1) (Rambaut and Drummond 2003). The consensus tree was constructed in TreeAnnotator (v1.4.8) and was visualized in the program FigTree (v1.2.2) (Drummond and Rambaut 2007).

### Biogeographic analysis

For ancestral-area reconstruction, each individual was assigned to one of the four phytogeographic regions suggested by Subramanyam and Nayar (1974) according to sampling location. The approximate boundaries of these zones are shown in the Figure 1. These regions are referred as biogeographic subdivisions. Ancestral-area reconstruction was done using Statistical Dispersal–Vicariance analysis (S-DIVA) (Yu et al. 2010). This program statistically evaluates the alternative ancestral ranges at each node in a tree accounting for phylogenetic uncertainty and uncertainty in DIVA optimization (Nylander et al. 2008; Harris and Xiang 2009; Yu et al. 2010). Bayesian phylogenetic trees for S-DIVA were obtained from the BEAST output file generated for divergence date analysis. Typically, in DIVA analysis all members of a species are assigned to the same region/regions depending on the distribution of the species. Here, we have assigned individuals of a species to a region based on the sampling location. This approach was taken to infer the ancestral area of a given species and also to detect and date range expansion events.

### Hypothesis testing

We used a Shimodaira–Hasegawa (SH) test to evaluate if the best tree was significantly different from trees based on S2 and S3 (Shimodaira and Hasegawa 1999). To this end constraint trees that are consistent with these scenarios were obtained using the program Mesquite (v 2.73) (Maddison and Maddison 2010). In the case of S2, species from south and north of PG were placed in two separate clades, and for S3, species were assigned to four different clades corresponding to the four biogeographic subdivisions. These constraint trees were then used to derive phylogenetic tress that are consistent with S2 and S3 in PAUP* through likelihood heuristic search (Swofford 2002). To examine the support for the S2 and S3, the likelihood scores of these constrained maximum likelihood (ML) trees were compared with unconstrained ML tree via one-tailed SH log-likelihood ratio test, as implemented in PAUP*, with full optimization resampling and 10,000 bootstrap replications.

## Results

### Biogeographic analysis

**Figure 2:**
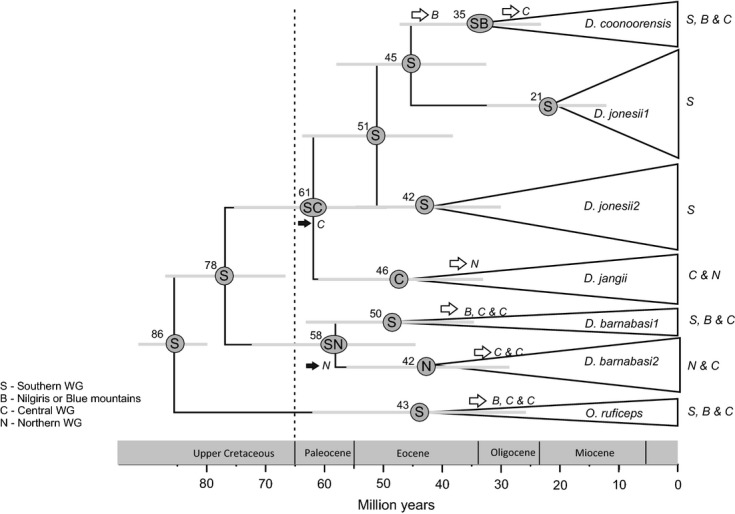
A time tree of genus *Digitipes* from the WG. Numbers indicate age of various nodes along with credible intervals (gray bars). Also shown are ancestral areas for various nodes (circles and ovals) and dispersal events (arrows). White arrows represent species range expansions and black arrows represent dispersals at the higher level of the phylogeny. Vertical dotted line shows K–T boundary.

Ancestral-area reconstruction in Statistical Dispersal–Vicariance analysis (S-DIVA) generated one optimal reconstruction involving 13 independent dispersal events with 100% support at all nodes (Fig. 2) (Yu et al. 2010). Of these 13 dispersal events, 11 explained the species’ range expansions while the remaining two dispersals were at a deeper level in the phylogeny. The ancestral areas of the two earliest nodes in the phylogeny, including the root node, were in the SWG. These two early dispersal events were from the SWG to the CWG and NWG (black arrows in Fig. 1). Among the 11 range expansion–dispersal events, seven were from the SWG to CWG and Nilgiris, whereas the rest were between CWG and NWG (white arrows in Fig. 1). Thus, a majority of the dispersal events (9/13) were from the SWG to other areas of the WG.

### Divergence date estimation

A Bayesian approach with a relaxed molecular clock model was employed to estimate divergence dates for various nodes (Drummond and Rambaut 2007). The nodes discussed here had effective sample size >200 in Bayesian divergence estimation. The 11 range expansion–dispersal events occurred sometime after 50 mya and span the Eocene-Miocene epochs (Table 1). These dates and the associated credible intervals (CI) were in the post-volcanic period. The diversifications following the two early dispersal events from SWG to CWG and NWG occurred around 58–61 mya also in the post-volcanic period in the Paleocene. As listed above, Scenarios S2 and S3 were not congruent with the current phylogenetic relationships among the *Digitipes* species. Additionally, the SH test suggested that the S2 and S3 were significantly different from the observed *Digitipes* phylogenetic tree (*P* < 0.05). Thus, the species-level phylogeny did not correspond to the PG or the biogeographic subdivisions of the WG.

**Table 1:** Ancestral area, ESS support, and divergence time for major *Digitipes* lineages based on BEAST analysis on the mtDNA

Node	Ancestral area	Mean (my)	CI	ESS
Genus *Digitipes* in WG	SWG	86	79–91	2102
Except *O. ruficeps*	SWG	78	65–86	975
*D. jangii* + *D. jonesii 1 &* 2 + *D. coonoorensis*	SWG & CWG	61	48–75	584
*D. jangii*	CWG	46	32–60	661
*D. barnabasi 1 – D. barnabasi* 2	SWG & NWG	58	43–71	435
*D. barnabasi* 1	SWG	50	35–63	538
*D. barnabasi* 2	NWG	42	28–55	565
*D. jonesii*	SWG	21	12–32	791
*D. jonesii*	SWG	42	30–54	726
*D. coonoorensis*	SWG & Nilgiris	35	24–48	814
*O. ruficeps*	SWG	43	25–60	750

## Discussion

This is the first historical biogeographical study from the WG, wherein molecular phylogenies, divergence date estimations, and ancestral-area reconstructions have been employed to study current patterns of species distributions and richness. Ancestral-area reconstruction (S-DIVA) retrieved SWG as the ancestral area for the WG lineage of *Digitipes*. The earliest dispersal events in the phylogeny were from the SWG to the CWG and NWG. Subsequent dispersals resulting in species range expansions were also largely from the SWG to other parts of the WG. Additionally, the basal species in the phylogeny was from SWG and current species diversity and endemicity were highest in the SWG. These results suggest that SWG might have been the center of origin for the extant WG *Digitipes* species. Interestingly, molecular dating suggests that 11 of the 13 dispersal events occurred in the Eocene- Miocene, 15–50 my after the Late Cretaceous volcanism. More importantly, two of the earliest dispersal events from SWG also appear to have occurred not long after the period of volcanism in the Early Paleocene. The Late Cretaceous volcanism generated a large volume of flood basalt over much of Northern PI (including NWG) and caused extensive extinction of its flora and fauna (Samant and Mohabey 2009). However, by the Early Paleocene much of the flora was reestablished (Prasad et al. 2009). Thus, it is likely that the SWG served as a refuge during Cretaceous volcanism from which species dispersed northward with the advent of suitable conditions. In the case of the genus *Digitipes*, northward dispersal also commenced in the Paleocene, and by Eocene-Miocene the current species diversity was established.

The PG did not emerge as a geographic barrier in the higher level phylogeny of the genus *Digitipes*. However, detailed phylogeographic studies might reveal the role of this gap in shaping genetic structure of species at a finer scale. For example, within *D. coonoorensis* the PG seems to have played a role as a geographic barrier as it had two reciprocally monophyletic groups across the gap. Testing the role of PG or any other barrier requires further fine-scale sampling of *Digitipes* species in the WG. *Digitipes* species did not form clusters according to the four biogeographic subdivisions, rather CWG, NWG, and NH species were nested within a larger clade of the SWG species.

### Implications for phylogenetic niche conservatism hypothesis

Results from our study have implications for studies on the origin of tropical diversity, such as those that invoke phylogenetic niche conservatism (PNC) (Wiens and Graham 2005; Wiens et al. 2010). According to PNC, species will disperse primarily within climatically similar areas rather than to areas with very different climate, even if those areas are geographically close (Wiens and Donoghue 2004; Wiens and Graham 2005). PNC also provides explanations of species richness for a given area by making two predictions: (1) a group with high tropical species richness originated in tropical regions (as shown by ancestral-area reconstruction on a phylogeny) (Wiens and Donoghue 2004), and (2) a significant positive relationship between the amount of time that a group has been present in each region and the number of species in each region (Stephens and Wiens 2009). In the case of *Digitipes*, a taxon confined to wet evergreen forest, it has failed to establish in the adjacent dry areas and has dispersed only into climatically similar areas over evolutionary time. Additionally, the SWG has more *Digitipes* species than other regions, indicating that there has been longer time for speciation in the SWG as compare with CWG, NH, and NWG.

Hence, this analysis affirms that historical processes like evolutionary origin and effects of refuge also need to be considered while conducting community ecology studies looking at species dynamics in the WG. Furthermore, phylogenetic relationships along with robust divergence date estimates can provide insights into the patterns and underlying causes of the historical biogeography of lineages. This dated molecular phylogeny of WG *Digitipes* species will be useful for future comparative historical biogeography and diversification studies in the WG. The Late Cretaceous refugial scenario that is invoked here to explain current species distributions and richness may be mostly relevant to ancient endemic lineages, and may not apply to groups that have recently dispersed into India or that have diverged recently. Importantly, the fascinating biogeographical scenario explored here needs to be tested in other such groups from WG.

Taken together these results suggest that SWG was a refuge for *Digitipes* species during Cretaceous volcanism, and in the post-volcanic periods the taxa expanded their ranges to other areas of the WG. Interestingly, neither the PG nor biogeographic subdivisions appeared to have influenced branching pattern at the species level. In addition, this study highlighted the fact that historical processes could potentially influence species diversity and distribution. Inferences from phylogenetic relationships, estimation of divergence time, and reconstruction of ancestral areas constitute effective tools in understanding the historical biogeography of the WG, and this phylogeny could be potentially used in future comparative studies on the WG taxa.
